# *Staphylococcus aureus* nasal carriage before breast reconstruction: antibiotic resistance, biofilm formation, and virulence genes—a single center *in vitro* observation

**DOI:** 10.3389/fmicb.2025.1610739

**Published:** 2025-06-19

**Authors:** Sylwia Jarzynka, Anna Koryszewska-Bagińska, Tomasz Nowikiewicz, Anna Szczepańska, Gabriela Olędzka, Maria Szymankiewicz

**Affiliations:** ^1^Department of Medical Biology, Medical University of Warsaw, Warsaw, Poland; ^2^Department of Surgical Oncology, Nicolaus Copernicus University in Toruń, Collegium Medicum in Bydgoszcz, Bydgoszcz, Poland; ^3^Department of Clinical Breast Cancer and Reconstructive Surgery, Prof. F. Łukaszczyk Oncology Centre, Bydgoszcz, Poland; ^4^Department of Microbiology, Prof. F. Łukaszczyk Oncology Centre, Bydgoszcz, Poland

**Keywords:** methicillin-sensitive *Staphylococcus aureus* (MSSA), screening nasal swabs, breast reconstruction, adhesion genes, antibiotic-resistant genes, virulence factors, biofilm formation, exopolysaccharide production

## Abstract

**Introduction:**

Infections caused by *Staphylococcus aureus (S. aureus)* in patients undergoing mastectomy followed by breast reconstruction present significant therapeutic challenges. Studies suggest that *S. aureus* may be transmitted from nasal carriage, potentially leading to postoperative infections. However, knowledge regarding the potential pathogenicity of *S. aureus* nasal carriage strains in women undergoing breast reconstruction in Poland remains limited. This study aimed to characterize *S. aureus* isolates obtained from screening nasal swabs.

**Methods:**

A total of 33 methicillin-sensitive *S. aureus* (MSSA) isolates were analyzed. These strains exhibited a high prevalence of genes encoding adhesion and antibiotic resistance. The most frequently detected virulence genes included *sarA* (100%), an activator of protein A; *cna* (100%), encoding collagen adhesin; *blaZ* (100%), associated with *β*-lactamase production; the *icaADBC* operon (82–100%), responsible for extracellular polysaccharide synthesis and intracellular adhesion; and bap (36%), encoding a surface-associated biofilm protein.

**Results:**

Most isolates (79–100%) demonstrated a strong capacity for biofilm formation and exopolysaccharide production, confirmed by independent methods. Notably, all strains (100%) remained susceptible to ciprofloxacin at increased exposure levels. RAPD analysis revealed low genetic diversity among the isolates.

**Discussion:**

Our findings indicate that *S. aureus* isolates from nasal carriers undergoing breast implantation exhibit antibiotic resistance, biofilm-forming ability, and harbor multiple virulence genes. Early detection of *S. aureus* colonization via nasal swab screening may be crucial for managing infection risk in patients undergoing breast reconstruction.

## Graphical abstract

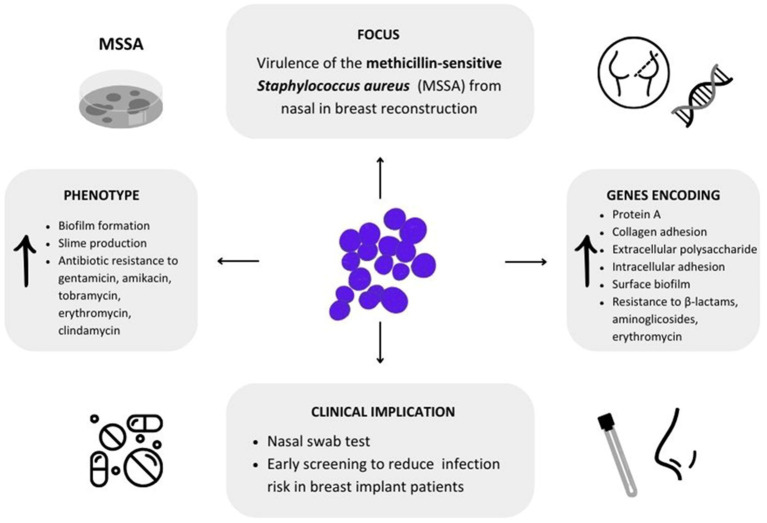

## 1 Background

*Staphylococcus aureus* (*S. aureus*) is commonly found in the human microbiome of the upper pulmonary tract and skin. Persistent nasal carriage of *S. aureus*, found in 10–30% of the general population, is a risk factor for many types of infections (Sakr et al., 2019). The prevalence of *S. aureus* nasal carriage infections is observed in a wide spectrum of diseases, for example, in patients with osteoarthritis, degenerative joint disease, rheumatoid arthritis, surgical wound infections (SWIs), chronic autoimmune inflammatory disease (Asai et al., 2022; Lemaignen et al., 2018), and surgical site infections (SSIs). The cases of peri-implant surgery infections may affect 18–45.2% of patients, caused primarily due to *S. aureus*; however, most etiological agents are *Staphylococcus epidermidis* (29%) and Gram-negative rods (24–54.75%) (Flouchi et al., 2021; Michalopoulos et al., 2022; Mukhtar et al., 2009; Viola et al., 2014). Nasal carriage of *S. aureus* is related to 20–42% of breast implant-associated infections (BIAIs) (Michalopoulos et al., 2022; Mukhtar et al., 2009; Viola et al., 2014; Nahabedian et al., 2003; Duran Ramirez et al., 2022; Tobias et al., 2023).

Current literature lacks studies focused exclusively on MSSA; most available analyses primarily emphasize methicillin-resistant *Staphylococcus aureus* (MRSA). However, MSSA accounts for 30.6% of breast implant infections, while MRSA is responsible for only 3.5% (Franchelli et al., 2018). The potential differences in pathogenicity and virulence between MRSA and MSSA strains remain unresolved, although proteomic changes have been suggested as contributing factors (Rozgonyi et al., 2007; Tu et al., 2021). Some studies even question whether MSSA might be more pathogenic than MRSA in clinical settings (Bride et al., 2019). Although MSSA is a recognized cause of surgical site infections (SSIs), its specific role via nasal carriage in implant-based breast reconstruction remains poorly understood. High-level MSSA nasal carriers have a 3–6 fold higher risk of healthcare-associated infections than non-carriers or low-level carriers (Tsang et al., 2018). Notably, most of these infections are endogenous, originating from the patient’s microbiota (Long et al., 2024; Wenzel, 2019). MSSA nasal carriage is becoming increasingly common, particularly among oncological patients; however, data on its characteristics in Polish women undergoing breast reconstruction are limited.

This study aims to address this knowledge gap by investigating the role of MSSA nasal carriage in SSIs following breast reconstruction in a Polish cohort. Moreover, the patient’s clinical conditions influence the surgical procedure, which may affect infection risk. For example, lymph node dissection has been associated with a 6.29-fold increased risk of implant infection compared with procedures without lymph node removal (Rouquette et al., 2015). SSIs may also affect the phenotypic expression and virulence gene profiles of *S. aureus* strains. These strains possess multiple virulence factors, notably a strong capacity for biofilm formation, which enhances adhesion to collagen and materials commonly used in breast implants. Infection can lead to inflammation, pain, and swelling, and in severe cases, may necessitate implant removal.

BIAIs are a major complication following mastectomy with reconstruction and often require antibiotic therapy or implant removal (Duran Ramirez et al., 2022; Kathju et al., 2024; Lohmeyer et al., 2021). Anti-MSSA antibiotics have been associated with a reduced risk of SSIs both in patients undergoing mastectomy alone and those with immediate reconstruction (Warren et al., 2022). Nonetheless, MSSA can exhibit antibiotic resistance, such as macrolide-lincosamide-streptogramin B (MLSB) resistance or vancomycin intermediate/resistant phenotypes (VISA/VRSA) (Warren et al., 2022; Kengne et al., 2024). Antibiotic prophylaxis, including MSSA eradication, has been shown to reduce SSI risk post-mastectomy. Clinical strategies for nasal decolonization include mupirocin nasal ointment, chlorhexidine body washes, vancomycin, triple antibiotic pocket irrigator (TAPI), and doxycycline-coated silicone implants (Lisa et al., 2024; Duran Ramirez et al., 2023; Hemmingsen et al., 2024; Baker et al., 2020; Gofstein-Hayuth et al., 2023). However, increasing resistance to mupirocin highlights the need for novel decolonization agents (Sakr et al., 2019; Labrecque et al., 2023). Preoperative screening for *S. aureus* nasal carriage, including both MSSA and MRSA, followed by appropriate antibiotic prophylaxis, can help reduce the incidence of BIAIs and shorten hospital stays (Humphreys et al., 2016). To address these clinical concerns, multicenter randomized trials are necessary to establish clear guidelines for screening and treatment of MSSA and MRSA nasal carriage in patients undergoing breast reconstruction (Hart et al., 2017; Papa et al., 2021). Finally, maintaining proper hygiene of the hands and surgical site is essential for women with breast implants to reduce the risk of *S. aureus*-related infections (Zhao et al., 2021).

This study aimed to characterize *S. aureus* strains isolated from the nasal carriage of Polish patients qualified for implant breast reconstruction, with a focus on the pathogenicity potential of these strains.

### 1.1 Experiment design

Screening of *Staphylococcus aureus* nasal carriage was performed on all patients undergoing treatment at the Department of Clinical Breast Cancer and Reconstructive Surgery at the Oncology Center, Prof. Franciszek Łukaszczyk Memorial Hospital in Bydgoszcz, who were eligible for breast reconstruction from June 2020 to August 2021. The patients had undergone mastectomy and post-mastectomy reconstruction. A total of 132 reconstructions among 124 patients were performed. All patients were screened for *S. aureus* nasal carriage before admission to the hospital. Clinical samples were collected within 30 days before hospitalization, according to standard laboratory protocols and following the ethical standards of the institutional and/or national research committee, along with the 1964 Helsinki declaration and its later amendments or comparable ethical standards. This study was approved by the Bioethics Committee of the Collegium Medicum UMK in Bydgoszcz (registration number KB 578/2021). A study was provided on clinical isolates from a retrospective collection collected over June 2020 and August 2021.

### 1.2 Material—study group

In the study, positive nasal screening was observed in 31 women (25%) before breast reconstruction. A total of 33 *S. aureus* isolates were obtained from these 31 patients.

The isolates were stored in tryptic soy broth (BioMérieux, France) with 20% (v/v) glycerol (Merck, Germany) and frozen at −70 ± 2°C until further pathogenicity analysis. Each patient contributed one isolate, whereas two patients provided two isolates each due to delayed surgeries.

**Figure 1:**
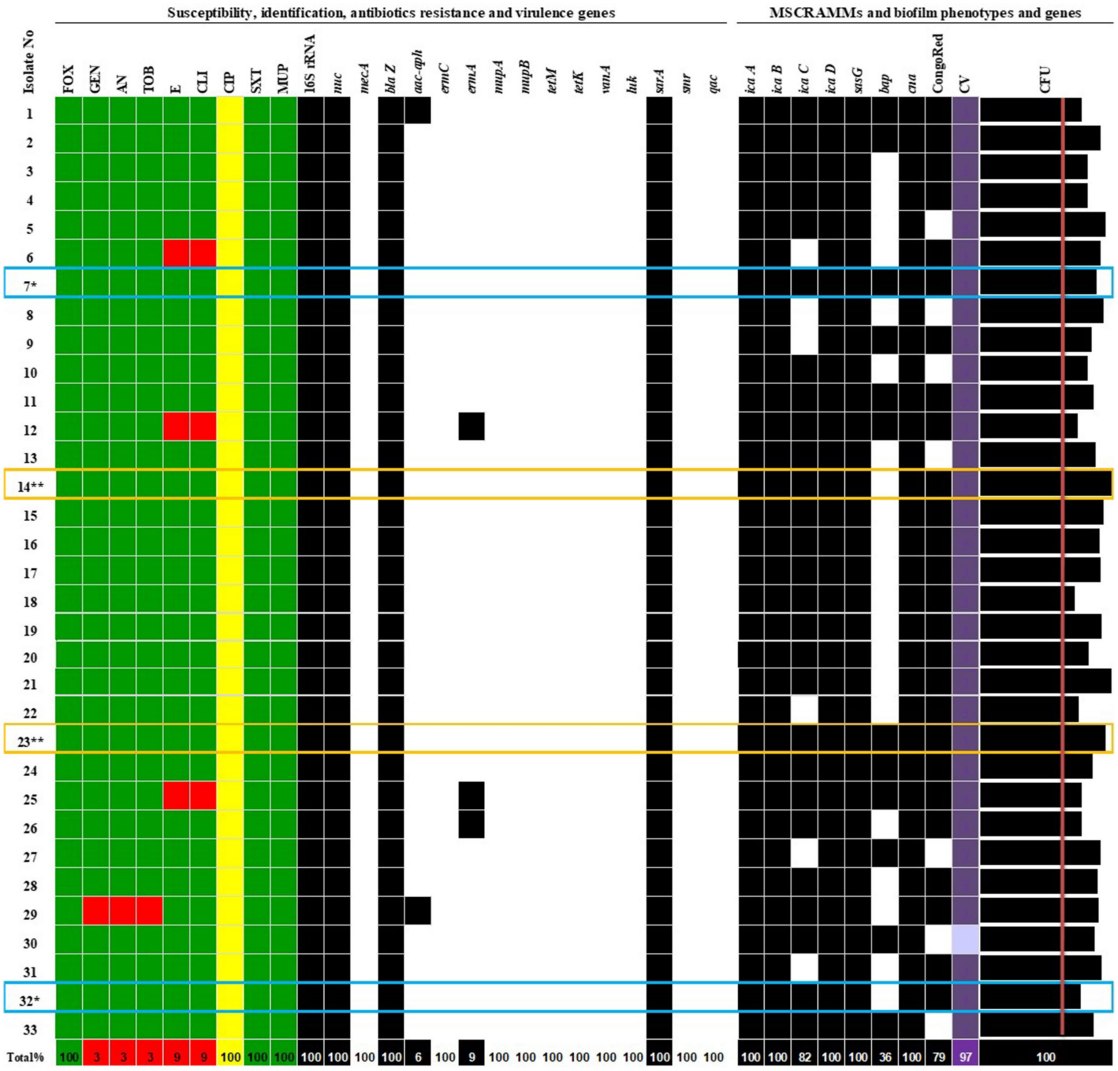
Phenotypic and genotypic characterization of *S. aureus* isolates. More than one *S. aureus* isolate from patient: 7* / 32* and 14** / 23**. Antibiotics: FOX, cefoxitin; GEN, gentamicin; AN, amikacin; TOB, tobramycin; E, erythromycin; CLI, clindamycin; CIP, ciprofloxacin; SXT, trimethoprim/sulfamethoxazole; MUP, mupirocin; MSSA, methicillin-sensitive *Staphylococcus aureus*. Genes: *icaA*, *icaB*, *icaC*, *icaD, sasG, bap, cna*, biofilm formation and adhesion; *luk, smr, qac, sarA*—encoding Panton-Valentine leukocidin (PVL), chlorhexidine and quaternary ammonium resistance, antiseptic resistance, an activator of protein A; *mecA, blaZ, aac-aph, ermC*, *ermA*, *mupA*, *mupB, tetM, tetK*, and *vanA*—encoding resistance to methicillin, *β*-lactams, aminoglycosides, erythromycin, mupirocin, tetracycline, and vancomycin. 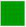
S, sensitive to antibiotics; 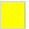
I, susceptible, increased exposure; 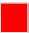 R, resistant; 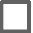 0, lack of gene; 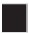 1, gene detection. CongoRed—exopolysaccharide synthesis phenotype; CV—biofilm staining by crystal violet and evaluated by spectrophotometric method, the mean values of absorbance for biofilm mass: light purple—moderate biofilm producer, purple—strong biofilm producer; CFU—biofilm cell count of averaged values in Log CFU/mL > initial concentration (red line—6.0 Log CFU/mL).

In one case, a second *S. aureus* was obtained approximately 1 month after the first isolate (14** and 23**, respectively). In another case, a second isolate (number 32*) was isolated 8 months after the first (number 7*) (Figure 1). The reason for repeated sampling was the postponement of surgical procedures for clinical reasons and a second reconstruction was planned (replacement of the expander with an implant).

## 2 Methods

*Retrospective analysis* presented in this study is based on a database from the Department of Microbiology, Prof. F. Łukaszczyk Oncology Centre in Bydgoszcz, regarding standard tests for phenotypic identification of microorganisms and describing antibiotic sensitivity.

### 2.1 Identification

The samples were processed using culture-based methods on a Staph/Strep Selective Medium (Oxoid Deutschland GmbH, Wesel, Germany) under aerobic conditions at 35 ± 2°C. After 24 h, suspected *S. aureus* isolates were identified using a coagulase test, the commercial Dry Spot Staphytest Plus (Oxoid Limited, Basingstoke, Hampshire, England), and matrix-assisted laser desorption/ionization time of flight mass spectrometry (IVD MALDI Biotyper Smart System, microflex LT/SH smart, Bruker Daltonik, Bremen, Germany).

### 2.2 Antimicrobial typing

Cefoxitin, gentamicin, amikacin, tobramycin, erythromycin, clindamycin, ciprofloxacin, trimethoprim/sulfamethoxazole, and mupirocin were selected for susceptibility testing, determined using the disk diffusion method on Mueller–Hinton agar (BioMérieux, France) following the European Committee on Antimicrobial Susceptibility Testing (EUCAST). The results were interpreted according to the EUCAST breakpoint tables v. 15.0 from 2025-01-01 (The European Committee on Antimicrobial Susceptibility Testing, 2025). *S. aureus* ATCC 29213 MSSA and ATCC 43300 MRSA were included as quality controls.

### 2.3 MRSA screening

Screening swabs taken from the patients were inoculated onto Brilliance MRSA 2 agar (Thermo Fisher Scientific, UK) for MRSA detection. All agar plates were read after 18–24 h of incubation at 35 ± 2°C, following the manufacturer’s instructions. Affiliation to the MRSA/MSSA group was further confirmed using the PCR Xpert *S. aureus* Nasal Complete Assay (Cepheid, US), which detects specific sequences of staphylococcal protein A (*spa* gene), the methicillin/oxacillin resistance (*mecA*), and the staphylococcal cassette chromosome (SCC*mec*) inserted into the chromosomal *attB* site.

*Experimental analyses* of virulence factors and genes encoding them were performed at the Department of Medical Biology, Medical University of Warsaw.

### 2.4 Characterization of exopolysaccharide (slime) production

Exopolysaccharide production (commonly referred to as the slime phenotype in earlier literature) was assessed using Congo Red Agar (CRA), prepared with 0.8 g of Congo Red (Merck, Germany), 36 g of sucrose (Chempur, Poland), and 1.5% agar, and Brain Heart Broth (Thermo Fisher Scientific, UK) (Freeman et al., 1989; Arciola et al., 2001). After 72 h of aerobic incubation at 37°C, colonies were evaluated based on morphology: black/dark, rough, and dry colonies were classified as exopolysaccharide-positive (indicative of biofilm matrix synthesis), while smooth, red/Bordeaux-colored colonies were classified as negative.

### 2.5 Biofilm phenotypic qualitative and quantitative assays

Biofilm formation was assessed using a crystal violet staining assay (Stepanovic et al., 2000). Bacterial suspensions in Luria–Bertani (LB) broth (Merck, Germany) were adjusted to 10^6^ CFU/mL and added to sterile 96-well flat-bottomed polystyrene microplates (200 μL per well) (Kartell, Italy). Plates were incubated for 48 h at 35 ± 2°. The negative control wells contained LB broth only. After incubation, the wells were stained with 0.15% crystal violet (Merck, Germany) for 15 min at room temperature. Excess day was removed, and the bound dye was solubilized in 96% ethyl alcohol (Chempur, Poland). Absorbance was measured at 570 nm using a Synergy HTX multi-mode reader (BioTeK Instruments, USA). Biofilm production was analyzed by comparing the absorbance values for negative (ATCC29213) and positive (ATCC12600) biofilm producers to those of a control without bacteria. The classes were defined as follows: An isolate < A control—non-biofilm producer, A control < A isolate < 2 × A control—weak producer, 2 × A control < A isolate < 4 × A control—moderate, and 4 × A control < A isolate, and strong producer. This experiment was repeated four times independently.

For the quantitative assay, biofilms were grown under identical conditions. After the supernatants were removed, the biofilms were resuspended in 150 μL of PBS (Thermo Fisher Scientific, UK) by scraping. Serial 10-fold dilutions were prepared up to 10^−5^, and 10 μL of aliquots were plated on LB agar. Log CFU/mL was calculated after overnight incubation at 35 ± 2°C. The experiment was performed in two independent replicates.

### 2.6 DNA extraction and PCR

Genomic DNA from *S. aureus* isolates was extracted using a Genomic Mini DNA Kit (A&A Biotechnology, Poland) according to the manufacturer’s instructions, with the addition of lysostaphin solution (10 mg/mL) and lysozyme (4,000 μg/mL) (A&A Biotechnology, Poland). DNA quality and quantity were assessed spectrophotometrically (NanoDrop; DeNovix Ds-11, US). Residual RNA was removed by RNase A digestion (Blirt S.A., Poland).

PCR reactions were performed in a Mastercycler Personal (Eppendorf, Germany) using primers listed in Table 1. The length of PCR products was determined by electrophoresis on a 1.5% (w/v) agarose gel stained with 0.5 μg/mL ethidium bromide (Merck KGaA; Darmstadt, Germany). DNA patterns were visualized and analyzed using ImageLab software (Bio-Rad, Inc., US).

**Table 1:** Primers used in study.

Gene	Sequence (5′-3′)	Product size (bp)	References
*16S rRNA*	AACTCTGTTATTAGGGAAGAACA	756	McClure et al. (2017)
	CCACCTTCCTCCGGTTTGTCACC		
*nuc*	GCGATTGATGGTGATACGGTT	279	McClure et al. (2017)
	AGCCAAGCCTTGACGAACTAAAGC		
*icaA*	ACACTTGCTGGCGCAGTCAA	188	Kouidhi et al. (2010)
	TCTGGAACCAACATCCAACA		
*icaB*	CCCAACGCTAAAATCATCGC	1,080	Gowrishankar et al. (2016)
	ATTGGAGTTCGGAGTGACTGC		
*icaC*	CATGAAAATATGGAGGGTGG	1,000	Kouidhi et al. (2010)
	TCAAACTGATTTCGCCCACCG		
*icaD*	ATGGTCAAGCCCAGACAGAG	198	Gowrishankar et al. (2016)
	AGTATTTTCAATGTTTAAAGCA		
*sasG*	CGAGCTTTTCTAACCTTAGGTGTC	188	Reiter et al. (2012)
	ACCACAGGGTGTAGAAGCTAAATC		
*bap*	GCTGTTGAAGTTAATACTGTACCTGC	971	Cucarella et al. (2001)
	CCCTATATCGAAGGTGTAGAATTGCAC		
*cna*	AAAGCGTTGCCTAGTGGAGA	192	Montanaro et al. (1999)
	AGTGCCTTCCCAAACCTTTT		
*luk*	GCATCAAGTGTATTGGATAGCAAAAGC	443	Mkhize et al. (2021)
	ATCATTAGGTAAAATGTCTGGACATGATCCA		
*sarA*	CCCAGAAATACAATCACTGTG	720	Gowrishankar et al. (2016)
	AGTGCCATTAGTGCAAAACC		
*smr*	GCCATAAGTACTGAAGTTATTGGA	195	McClure et al. (2017)
	GACTACGGTTGTTAAGACTAAACCT		
*qac*	GCAGAAAGTGCAGAGTTCG	361	McClure et al. (2017)
	CCAGTCCAATCATGCCTG		
*blaZ*	ACTTCAACACCTGCTGCTTT	173	Mkhize et al. (2021)
	TGACCACTTTTATCAGCAAC		
*mecA*	GTAGAAATGACTGAACGTCCGATAA	310	Parvin et al. (2021); Zhang et al. (2004)
	CCAATTCCACATTGTTTCGGTCTAA		
*aac-aph*	TAATCCAAGAGCAATAAGGGC	227	Mkhize et al. (2021)
	GCCACACTATCATAACCACTA		
*ermC*	AATCGTCAATTCCTGCATGT	299	Lina et al. (1999)
	TAATCGTGGAATACGGGTTTG		
*ermA*	AAGCGGTAAACCCCTCTGA	190	Lina et al. (1999)
	TTCGCAAATCCCTTCTCAAC		
*tetM*	GTTAAATAGTGTTCTTGGAG	158	Aarestrup et al. (2000)
	CTAAGATATGGCTCTAACAA		
*tetK*	TTAGGTGAAGGGTTAGGTCC	360	Aarestrup et al. (2000)
	GCAAACTCATTCCAGAAGCA		
*mupA*	TATATTATGCGATGGAAGGTTGG	456	McClure et al. (2017)
	AATAAAATCAGCTGGAAAGTGTTG		
*mupB*	CTAGAAGTCGATTTTGGAGTAG	674	McClure et al. (2017)
	AGTGTCTAAAATGATAAGACGATC		
*vanA*	GGCAAGTCAGGTGAAGATG	713	Maharjan et al. (2021)
	ATCAAGCGGTCAATCAGTTC		

All primer syntheses were performed by the DNA Sequencing and Oligonucleotide Synthesis Laboratory (IBB PAN, Warsaw, Poland).

### 2.7 PCR confirmation of *S. aureus* and MSSA/MRSA

The genus *Staphylococcus* was confirmed using specific 16S rRNA primers, and *S. aureus* was differentiated from coagulase-negative staphylococci (CoNS) using *nuc* gene primers (Bissong and Ateba, 2020; McClure et al., 2017; Mkhize et al., 2021; Shortle, 1983). Resistance to methicillin/oxacillin was confirmed by detecting the *mecA* (McClure et al., 2017; Parvin et al., 2021; Zhang et al., 2004).

PCR was performed in 25 μL volumes consisting of 10 pM of each primer, 10 μM deoxynucleotides, 10 x buffer, MgCl_2_, HotStar Taq Plus DNA polymerase (Qiagen, Netherlands), or recombinant Taq DNA polymerase, and template DNA. All reagents, unless specified, were obtained from Thermo Fisher Scientific, UK. The PCR conditions were as follows: an initial denaturation phase at 94°C for 5 min, followed by 25–35 cycles of denaturation at 94°C for 30 s, annealing at 55°C for 30 s or 1 min, extension at 72°C for 1 min, and a final extension phase at 72°C for 10 min.

*S. aureus* ATCC29213 MSSA, ATCC25923 MSSA, ATCC12600 MSSA, and ATCC43300 MRSA strains were used as controls.

### 2.8 Molecular analysis of biofilm and adhesion genes

Molecular analysis of adhesive and biofilm-forming genes focused on genes involved in polysaccharide intercellular adhesin (PIA) biosynthesis, including *icaA*, *icaB*, *icaC*, and *icaD*, which are critical for extracellular polysaccharide synthesis and intracellular adhesion (Arciola et al., 2001; Bissong and Ateba, 2020; Barbieri et al., 2015; Khudor and Idbeis, 2022; Pinto et al., 2015; Reiter et al., 2012; Vancraeynest et al., 2004; Vasudevan et al., 2003). The *sasG* gene, encoding surface adhesin (Barbieri et al., 2015; Pinto et al., 2015; Reiter et al., 2012), and the *bap* gene, encoding a cell wall-associated surface protein (Bissong and Ateba, 2020; Vancraeynest et al., 2004; Cucarella et al., 2001; Tormo et al., 2005; Vautor et al., 2008), were also examined. Furthermore, the *cna* gene, a member of the Microbial Surface Components Recognizing Adhesive Matrix Molecules (MSCRAMMs), responsible for collagen binding, was also analyzed (Barbieri et al., 2015; Vancraeynest et al., 2004; Cavalcante et al., 2021; Montanaro et al., 1999).

All PCR reactions were prepared as described above, using the same components in a total volume of 25 μL. The amplification of biofilm genes was performed as follows: 95°C for 5 min, 30–40 cycle of denaturation at 95°C for 20–30 s, annealing at a specific temperature for each gene for 30 s or 1 min, extension at 72°C for 1 min, and final extension at 72°C for 7 min.

PCR conditions for the *cna* gene were conducted with initial denaturation at 94°C for 4 min, followed by 35 cycles of denaturation at 94°C for 1 min, annealing at 54°C for 1 min, extension at 72°C for 1 min, and a final extension at 72°C for 10 min.

### 2.9 Detection of virulence and antibiotic resistance encoding genes

The genetic markers affecting *S. aureus* virulence and resistance profiles were identified based on a diverse array of genes associated with pathogenicity and antimicrobial resistance. Key virulence determinants included the *luk* gene (McClure et al., 2006), encoding Panton-Valentine leukocidin (PVL), associated with necrotic skin and soft tissue infections (Mkhize et al., 2021; Barbieri et al., 2015; Jarraud et al., 2002; Lina et al., 1999), and *sarA*, an activator of protein A (Gowrishankar et al., 2016).

Antiseptic resistance mechanisms were attributed to the *smr* encoding resistance to chlorhexidine and quaternary ammonium compounds (McClure et al., 2017; Datta and Huang, 2008), and *qac* genes (McClure et al., 2017; Noguchi et al., 2005).

For antibiotic resistance, several key genes were identified: aminoglycoside resistance genes *aac-aph* and *blaZ*, related to the production of *β*-lactamase and resistance to penicillin (Bissong and Ateba, 2020; Mkhize et al., 2021). The *vanA* gene is associated with vancomycin resistance (Bissong and Ateba, 2020; McClure et al., 2017; Al-Amery et al., 2019; Azimian et al., 2012; Girijan and Pillai, 2021; Maharjan et al., 2021; Vazquez-Rosas et al., 2021), and other genes, such as *tetK*, *tetM*, *ermA*, and *ermC*, are linked to tetracycline and erythromycin resistance, respectively (Mkhize et al., 2021; Aarestrup et al., 2000; Dong et al., 2020). Additionally, the *mupA* and *mupB* genes conferred mupirocin resistance (McClure et al., 2017; Seah et al., 2012).

All PCR reactions were prepared as described above, using the same components in a total volume of 25 μL Thermal cycling conditions were optimized for specific gene targets as follows: (1) *smr*, *qac*, and *mupA*/*mupB* 94°C 5 min, 10 cycles of 94°C 30 s, 60°C 30 s, 72°C 45 s, and next step 94°C 30 s, 25 cycles of 94°C 30 s, 50°C 30 s, 72°C 45 s, and 72°C 7 min; (2) *vanA* and *vanB* 94°C 5 min, 30 cycles of 94°C 30 s, *vanA* 54°C 1 min, *vanB* 50°C 45 s, 72°C 1 min, and 72°C 10 min; (3) *sarA* 94°C 4 min, 35 cycles of 94°C 1 min, 54°C 1 min, 72°C 1 min, and 72°C 10 min; (4) *luk* and *aac-aph* 95°C 5 min, 25 cycles of 95°C 30 s, 55°C 30 s, 72°C 45 s, and 72°C 7 min; (5) *blaZ* 95°C 5 min, 30 cycles of 95°C 30 s, 55°C 1 min, 72°C 1 min, and 72°C 7 min; (6) *ermA* and *ermC* 94°C 4 min, 35 cycles of 94°C 30 s, 53°C 30 s, 72°C 1 min, and 72°C 10 min; and (7) *tetK* and *tetM* 94°C 4 min, 35 cycles of 94°C 1 min, 55°C 1 min, 72°C 1 min, and 72°C 10 min.

*S. aureus* ATCC 29213 MSSA, ATCC 25923 MSSA, ATCC 12600 MSSA, and ATCC 43300 MRSA strains were used as quality control.

### 2.10 RAPD grouping method

The genetic relatedness of *S. aureus* carriage isolates was assessed using the Random Amplification of Polymorphic DNA PCR method (RAPD-PCR) with three short primers: AP-PCR1 (5′ GGTTGGGTGAGAATTGCACG 3′), AP-PCR7 (5′ GTGGATGCGA 3′), and ERIC2 (5′ AAGTAAGTGACTGGGGTGAGCG 3′) (Barbieri et al., 2015; van Belkum et al., 1995). These primers generated distinct RAPD-PCR banding patterns for each strain, reflecting their genetic diversity.

Each 25 μL reaction contained: 2.5 μL of 10 pM each primer, 2.5 μL dNTP Mix 10 mM, 2.5 μL 10 x buffer with MgCl_2_, 0.15 μL TaKaRa Ex Taq (all reagents were obtained from Takara Bio Inc., Japan), and 2.0 μL of DNA. The amplification was as follows: 94°C for 4 min, 35 cycles of denaturing at 94°C for 1 min, annealing at 25°C for 1 min, extension at 72°C for 7 min, and final extension at 72°C for 7 min.

DNA fingerprint patterns were visualized on agarose gel and analyzed using ImageLab software (Bio-Rad, US). Genetic distances were calculated, and a dendrogram was constructed using the genetic distance and neighbor-joining algorithm by PyElph 1.4 free software (PyElph by Pavel A. and Vasile C., USA) (Pavel and Vasile, 2012).

### 2.11 Statistical analysis

Statistical analyses were performed on pseudonymized data using IBM SPSS Pro 9 Statistics (IBM, USA, Medical University of Warsaw licence) and GraphPad Prism 9 (GraphPad Software, USA). The median, maximum, and minimum values were obtained, and the distribution of the parameters was assessed using Spearman’s correlation test. Graphics were created using Microsoft 365 (Microsoft Corporation, Redmond, USA, Medical University of Warsaw licence), and the correlation matrix was visualized by plotting with R software using online tools from http://www.sthda.com/english/rsthda/correlation-matrix.php by Kassambara (n.d.). Statistical significance for correlations (two-tailed) was set at ** *p < 0.01*. The following criteria were used to interpret correlation strength: weak *r_s_* > 0.3, moderate *r_s_* > 0.5, strong *r_s_* > 0.7, and very strong *r_s_* > 0.9.

## 3 Results

### 3.1 Antimicrobial susceptibility pattern

During 15-month observation period, positive nasal carriage of *S. aureus* was confirmed in 31 women (25%). A total of 33 isolates were identified as *S. aureus* species by routine laboratory testing using culture, biochemical, and mass spectrometry procedures. All isolates (100%) were confirmed as MSSA via Brilliance MRSA 2 agar culture, PCR Xpert SCC*mec* typing, and antimicrobial susceptibility testing.

All isolates were susceptible to cefoxitin (confirming MSSA phenotype), trimethoprim/sulfamethoxazole, and mupirocin.

Among the nine antibiotics tested, three isolates (9.1%) were fully sensitive to all tested drugs. All 33 *S. aureus* isolates (100%) were susceptible to ciprofloxacin with increased exposure, requiring higher drug concentrations for inhibition. Three isolates (9.1%) were resistant to clindamycin and erythromycin but were susceptible to ciprofloxacin with increased exposure. One isolate (3.0%) was resistant to three antibiotics, gentamycin, tobramycin, and amikacin, but was susceptible to ciprofloxacin with increased exposure. The identification and antimicrobial results are presented in Figure 1.

### 3.2 Biofilm formation and virulence gene distribution

**Figure 2:**
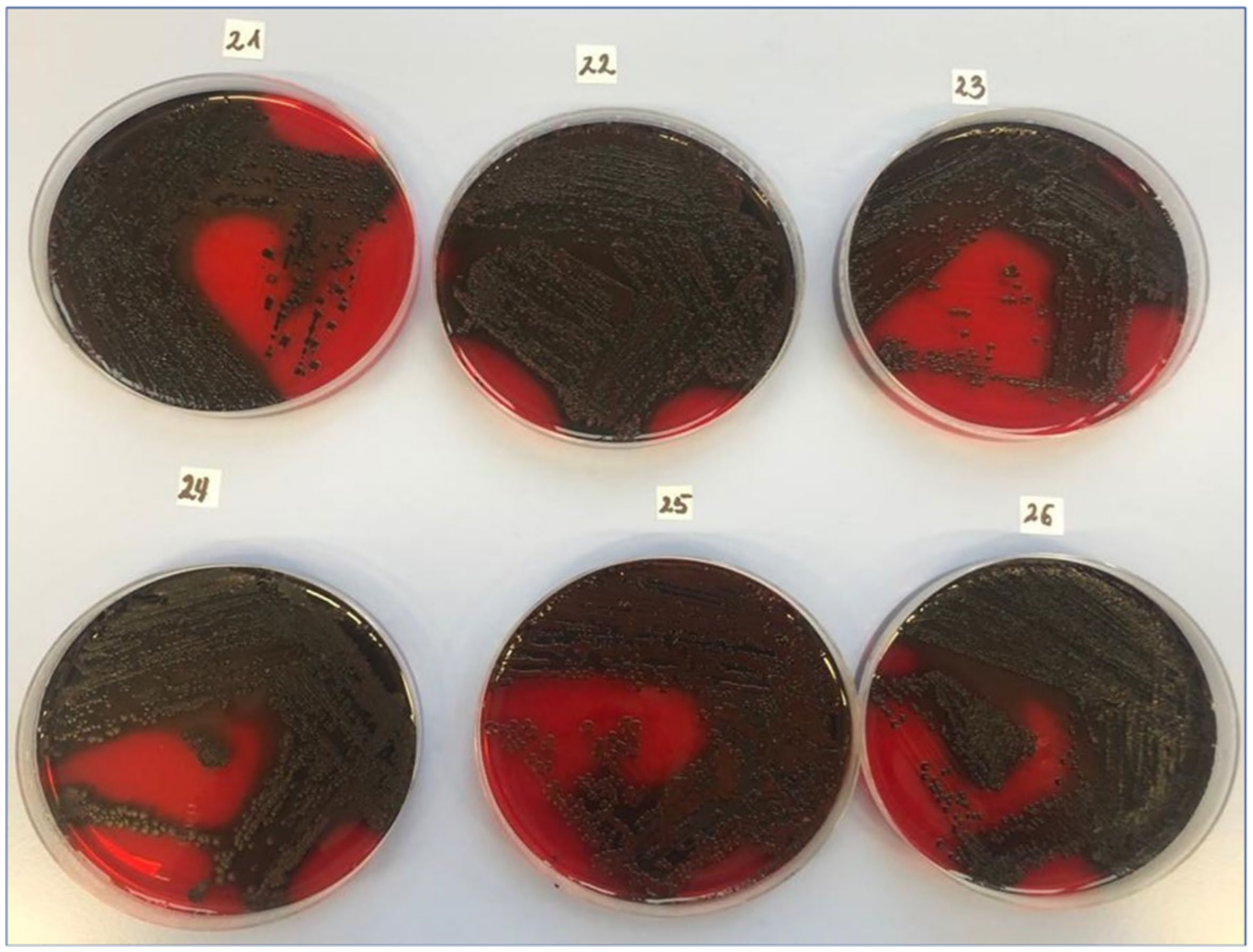
Slime production by clinical MSSA strains using the CRA method. CRA (Congo Red Agar), the test is prepared by adding 0.8 g Congo Red (Merck KGaA, Darmstadt, Germany). Exopolysaccharide-positive results (22–26 of the clinical isolates) were described as black or almost black colonies with a rough, very dry, crystalline consistency. Exopolysaccharide-negative colonies were smooth, red, or Bordeaux red with a darkening in the center.

The exopolysaccharide synthesis phenotype, assessed using Congo Red Agar, was detected in 79% of strains (Figure 2). Among the *S. aureus* study group, 97% of the strains were identified as strong biofilm producers using the qualitative method of crystal violet staining and spectrophotometric measurement. The quantitative assay confirmed biofilm formation in 100% of strains (data available in repository DOI 10.6084/m9.figshare.27910680).

Molecular analysis revealed that all isolates harboured *icaA*, *icaB*, *icaD*, *sasG*, and *cna*, involved in adhesion and biofilm formation (Figure 1, Table 2). Some isolates also had extra genes that varied between strains. The *icaC* gene was found in 82% of the isolates, and the *bap* gene was present in 36% of the strains. Notably, all isolates lacked the virulence determinants *luk* gene, which encodes Panton-Valentine leukocidin (PVL) associated with necrotic skin and soft tissue infection, the *smr* gene, encoding chlorhexidine and quaternary ammonium resistance, and the *qac* gene, involved in antiseptic resistance. The *sarA* gene, an activator of protein A, was detected in the genomes of all *S. aureus* strains. The distribution of virulence factors encoding genes is presented in Figure 1.

**Table 2:** Summary characteristics of MSSA in carriers undergoing breast reconstruction in the Polish cohort.

Category	Feature	Value
MSSA identification	*Staphylococcus aureus* (MALDI Biotyper, 16 s rRNA, *nuc*)	100%
	Protein A activator (*sarA*)	100%
	Methicillin resistance (*mecA*)	0%
Biofilm formation	Biofilm formation ability	100%
	Strong producer	97%
	Moderate producer	3%
	*icaA, icaB, icaD* genes	100% each
	Surface adhesin (*sasG*)	100%
	Collagen adhesin (*cna*)	100%
	*icaC* gene	82%
	Cell-wall-associated protein (*bap*)	36%
Exopolysaccharide production	Exopolysaccharide synthesis phenotype	79%
Antimicrobial susceptibility	Sensitive to cefoxitin, trimethoprim/sulfamethoxazole, and mupirocin	100%
	Sensitive to ciprofloxacin with increased exposure	100%
	Resistance to amikacin, tobramycin, gentamicin	3%
	MLSb resistance (erythromycin + clindamycin)	9%
	β-lactamase gene (*blaZ*)	100%
	Erythromycin resistance gene (*ermA*)	9%
	*ermC* gene	0%
	Aminoglycoside resistance gene (*aac-aph*)	6%
	Mupirocin resistance genes (*mupA, mupB*)	0%
	Tetracycline resistance genes (*tetM, tetK*)	0%
	Vancomycin resistance gene (*vanA*)	0%
Toxins/disinfectants	Panton-Valentine leukocidin gene (*luk*)	0%
	Chlorhexidine resistance gene (*smr*)	0%
	Antiseptic resistance gene (*qac*)	0%

### 3.3 Association between genotypic and phenotypic traits

Strains with full antibiotic sensitivity were characterized by *blaZ* and *aac-aph* resistance genes, high ability to form biofilms, and detection of *icaA-D*, *bap*, *sasG*, and *cna* virulence genes. Only 83.3% of the 30 isolates exhibiting increased exposure to ciprofloxacin carried the *icaC* gene, and 10% could not produce exopolysaccharides.

All 33 *S. aureus* isolates possessed blaZ, *sarA*, *icaA*, *icaB*, *icaD*, and *cna* genes. The *aac-aph* gene was detected in 6% of the strains, *ermA* in 9%, *tetM* in 3%, *icaD* in 82%, and *bap* in 36%. Detailed phenotypic and genotypic characteristics and antimicrobial susceptibility results are presented in Figure 1.

The study included two cases in which two nasal swabs were obtained from the same patient, resulting in the culture of different *S. aureus* isolates. In all cases where multiple cultures were obtained from the same patient, the virulence profiles of the isolates were consistent, showing MSSA with sensitivity to GEN, AN, TOB, E, CLI, STX, MUP, and FOX; susceptibility with increased exposure to CIP; and the presence of *blaZ, aac-aph, icaA, icaB, icaC, icaD, sarA, sasG*, and *cna* genes, as well as the ability to form biofilms. The only exception was the presence or absence of the *bap* gene (Figure 1). In the first case, the clinical condition of a patient undergoing breast reconstruction changed, leading to the collection of a second isolate (isolate number 32*, *bap* gene absent) 7 months after the first isolation (isolate number 7*, *bap* gene detected). In the second case, the patient was treated in the hospital when the first isolation was cultured (isolate number 14**, bap gene absent). Approximately a month later, a subsequent positive culture of *S. aureus* was obtained (isolate number 23**, *bap* gene detected).

### 3.4 Genetic relationship analysis of *S. aureus* nasal carriage isolates based on RAPD-PCR

**Figure 3:**
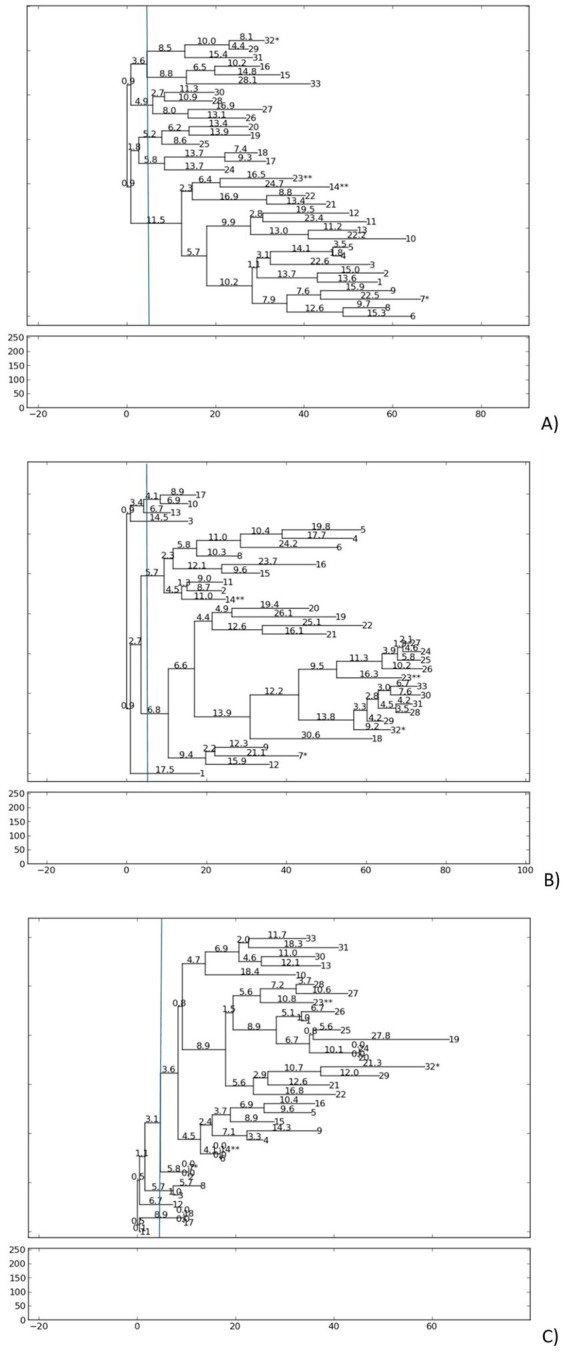
Phylogenetic dendrogram based on RAPD-PCR results for *S. aureus* strains, according to **(A)** PCR-ERIC2, **(B)** AP-PCR1, and **(C)** AP-PCR7 primers. The dendrogram was generated using the neighbor-joining method with PyElph software (USA). Genetic distances are demonstrated above the branches. More than one *S. aureus* isolate from patient: 7*/32* and 14**/23**.

The genetic relatedness of *S. aureus* isolates was assessed by RAPD pattern with ERIC2, AP-PCR7, and AP-PCR1 primers, separately. All primers revealed low genetic diversity among the 33 MSSA isolates, as shown by similar banding patterns (Figure 3, Supplementary Figure 1).

For ERIC2, amplification generated 4–8 fragments per (250 to 5,873 bp), with a polymorphic index (percentage of variable bands in RPAD profiles) of 5.2% and a genetic distance range (measure of divergence between isolates, where lower values indicate greater similarity) of 0.9 to 28.1. The isolates were clustered into five groups, with the largest separated into the next eight subgroups.

AP-PCR1 produced 5–9 fragments per isolate (250 to 5,880 bp), showing a polymorphic index of 5.5% and a genetic distance range of 0.9 to 30.6, forming six distinct groups at low genetic distance thresholds.

AP-PCR7 4–8 fragments per isolate (250 to 3,792 bp), with a p polymorphic index of 4.5% and a genetic distance range of 0.0 to 27.8, resulting in a dominant cluster of 25 isolates and five minor populations.

Double isolates from the same patients exhibited remarkable genotypic similarity. Isolates 23** and 14** from the same patient clustered together using ERIC2 and AP-PCR1. Isolates 7* and 32* in both RAPD reactions were grouped in separate clusters but exhibited low genetic distances (ERIC2 0.9, for AP-PCR1 2.7) despite differences in *bap* status. The low polymorphic indices and narrow genetic distance ranges confirm minimal divergence within the *S. aureus* population, suggesting clonal persistence in nasal carriers. Longitudinal isolates from the same patient retained near-identical genotypes over time, suggesting colonization by closely related strains.

### 3.5 Correlation of phenotypic factors and virulence gene detection

**Figure 4:**
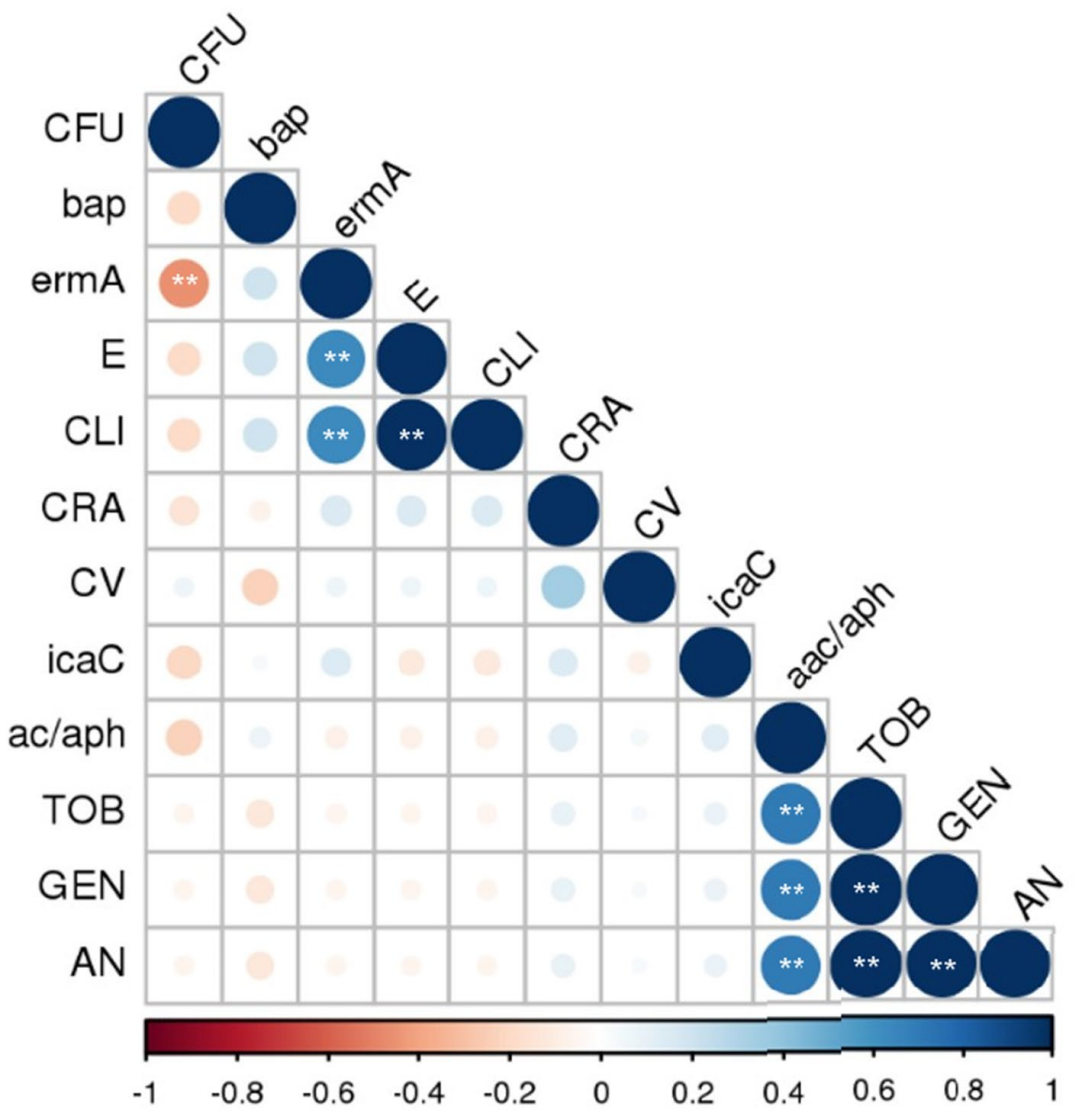
Correlation of the phenotypic and genotypic factors of MSSA virulence. MSSA, methicillin-sensitive *Staphylococcus aureus*; GEN, gentamicin; AN, amikacin; TOB, tobramycin; E, erythromycin; CLI, clindamycin; *icaC*, *bap*, biofilm formation and adhesion; *aac-aph*, *ermA*, encoding resistance to aminoglycoside and erythromycin, respectively. CRA (Congo Red Agar)—exopolysaccharide synthesis phenotype; CV—biofilm staining by crystal violet and evaluated by spectrophotometric method, the mean values of absorbance for biofilm mass; CFU—biofilm cells counting, an increase in the log value above the initial concentration Log (6.0 Log CFU/mL). Spearman correlation significance (two-tailed) was assumed at the level of ** *p* < 0.01. The following assumptions were applied to the correlation: weak r_s_ > 0.3, moderate r_s_ > 0.5, strong r_s_ > 0.7, and very strong r_s_ > 0.9.

Spearman’s correlation analysis revealed several significant findings (Figure 4). A significant positive correlation (*p < 0.01*) was observed between resistance to clindamycin and resistance to erythromycin (positive correlation; r_s_ = 1), which indicates MLS_b_ resistance to macrolides, lincosamides, and streptogramins. A significant positive correlation (r_s_ = 0.7) was found between the detection of the aminoglycoside resistance gene*s acc/aph* and tobramycin, gentamicin, and amikacin resistance within the same drug group. All aminoglycosides tested showed a statistically significant positive correlation with each other (r_s_ = 1). Among the other factors, significant (*p < 0.01*) but weak or moderate correlations were observed (data available in repository DOI 10.6084/m9.figshare.27910680). The correlation between *ermA* and *S. aureus* biofilm formation was weakly negative (r_s_ = − 0.41). The *ermA* gene also showed a moderate positive correlation with resistance to clindamycin (r_s_ = 0.63) and erythromycin (r_s_ = 0.63).

## 4 Discussion

*S. aureus* is a common cause of many surgical site infections (SSIs), including breast implant-associated infections in patients undergoing breast surgery (BIAIs). Clinical studies report that the occurrence of SSI is 3.5% within 90 days following mastectomy, 8.8% after mastectomy with immediate reconstruction, and up to 22% following planned reconstruction (Warren et al., 2022; Azouz et al., 2018; Szymankiewicz et al., 2023). A multicenter study from 2023 involving 5,004 patients identified preoperative *S. aureus* carriage, particularly in patients after mastectomy and neurosurgery, as well as those with a higher body mass index and non-removable implants, is associated with *S. aureus* SSIs, even postoperative bloodstream infections (BSIs) (Troeman et al., 2023). In our previous study, regarding 132 post-mastectomy breast reconstructions performed at a single oncology center in Bydgoszcz, Poland, we found a 5.3% infection rate. All infections were classified as deep incisional SSI (Szymankiewicz et al., 2023). In countries with high breast cancer rates, such as Poland, microbiological monitoring of this patient group is crucial not only for improving treatment outcomes, but also for the psychological state of patients, which may deteriorate in case of implant loss. Currently, breast cancer is the second leading cause of cancer-related deaths among women in Poland, after lung cancer. Data from the National Cancer Registry indicate that 17,500 women were diagnosed with breast cancer in Poland in 2020, with 7,500 deaths resulting from the disease (Wojciechowska et al., 2023). However, these numbers might be somewhat affected by the coronavirus disease of 2019 (COVID-19) pandemic, as the number of diagnoses in 2019 was higher, at 19,620 (National Cancer Registry in Poland, 2021). This finding suggests that the observed incidence may increase in the coming years. SSIs are among the most common healthcare-associated infections (HAIs) and significantly contribute to patient morbidity and healthcare costs.

Given this situation, more complications, including microbiological complications, can occur in these patients. The most concerning scenarios involve infections caused by antibiotic-resistant pathogens such as *S. aureus* MRSA. A study by Goudarzi et al. found that multidrug-resistant MSSA strains were present in 84% of the examined isolates from the nasal cavity of hospital employees. Authors observed strong biofilm formation and genetic relatedness of strains, which may suggest that MSSA clonal switching to the MRSA phenotype (Goudarzi et al., 2016). However, recent studies have indicated the increasing pathogenicity of antibiotic-sensitive strains, such as *S. aureus* MSSA. Their virulence is attributed to various factors, such as the production of mucus and biofilms and adhesins such as MSCRAMMs. The available literature offers limited data on the diversity of MSSA strains from nasal carriages in different patient groups, including those after mastectomy. Research has shown a high rate of antibiotic resistance among MSSA strains, with 92.2% resistant to penicillin, 45.3% to erythromycin, 6.3% to clindamycin, 7.8% to gentamycin, and 18.8% to ciprofloxacin (Li et al., 2022).

Some studies have reported multidrug-resistant MSSA in up to 84% of isolates (Goudarzi et al., 2020). In one such study, a highly pathogenic MSSA serotype ST239 characterized by strong biofilm formation, high persistence, and toxicogenicity was identified (Goudarzi et al., 2020). We observed full sensitivity for the nine antibiotics in only 9.1% of all isolates. Additionally, 100% of *S. aureus* isolates were susceptible to ciprofloxacin with increased exposure. Single isolates showed resistance to gentamicin, amikacin, tobramycin, erythromycin, clindamycin, and had genes encoding antibiotic resistance (*ermA, acc-aph*).

The literature reports that MSSA strains isolated from SSIs show generally high antibiotic sensitivity, with only slight resistance observed. Reported resistance rates include erythromycin (8.33%) (Flouchi et al., 2021; Horváth et al., 2020). All isolates were 100% sensitive to ciprofloxacin, lincomycin, tobramycin, and amikacin. Among MSSA strains from general infections, resistance was more varied: erythromycin (19%), ciprofloxacin (8%), clindamycin (8%), chloramphenicol (3%), gentamicin (data not specified), trimethoprim/sulfamethoxazole (8%), and tetracycline (28%) (Bride et al., 2019). These findings indicate that MSSA strains can also develop antimicrobial resistance, underlining the need for further studies to determine the clinical significance of this phenomenon.

Resistance to clindamycin was strongly positively correlated with resistance to erythromycin (*p < 0.01*) and moderately positively correlated with detection of the *ermA* gene (*p = 0.000*), which was predictable. A strong positive correlation between the detection of the gene encoding antibiotic resistance and the corresponding antibiotic was also observed for all tested aminoglycosides and the *acc-aph* gene (*p = 0.000*). In a study by Goudarzi et al. (2016) on *S. aureus* nasal carriage following breast reconstruction with expanders and implants, the presence of *ermC* and *ermA* was demonstrated in 33 and 3% of cases, respectively. The present study detected these genes in 0 and 9% of cases. Literature data indicate that *S. aureus* strains isolated from the intestines of oncological patients, including those with breast cancer, exhibit higher resistance to *β*-lactams and ciprofloxacin than those isolated from non-oncological patients (Kengne et al., 2024).

A comparison between MSSA isolates responsible for bone and joint infections and those from nasal colonization revealed a similar prevalence of *β*-lactamase (*blaZ*) production. This suggests that *blaZ*-positive MSSA strains implicated in postoperative infections were not necessarily selected for antibiotic prophylaxis (Trouillet-Assant et al., 2016). Similarly, in the study by Ngoi et al. (2021), a *blaZ*-positive MSSA strain was identified as the cause of a fatal bloodstream infection. Interestingly, core genome phylogeny revealed that this MSSA strain shared a common ancestry with a European MRSA clone (Ngoi et al., 2021). This raises the question: could a similar genotypic shift occur among *S. aureus* strains isolated from patients undergoing breast reconstruction? This remains an important issue for further investigation.

In this study, the *blaZ* gene, associated with the production of *β*-lactamase and resistance to penicillin, was detected in all *S. aureus* strains. The high detection rate of *blaZ* may be related to the production of β-lactamases, which is often the result of acquiring the *blaZ* gene in an inducible manner, meaning that it is expressed in the presence of a β-lactam antibiotic. This gene is frequently located on mobile elements such as transposons, insertion sequences, and plasmids (Li et al., 2022). The literature suggests that ciprofloxacin-resistant *S. aureus* has a higher potential for biofilm formation (Yasir et al., 2021; Kashef et al., 2020). However, this effect was not observed in our study. All strains with 100% ciprofloxacin susceptibility and increased exposure were biofilm producers. Nonetheless, some of these strains did not produce exopolysaccharides and lacked the *bap* and *icaC* genes, but possessed the *acc-aph, ermA* genes, which encode resistance to aminoglycosides, erythromycin, and tetracycline, respectively. In a study conducted by McClure et al. (2017), the *mupA* gene was found in all MSSA strains, whereas *mupB* was absent, and these strains were resistant to mupirocin. The emergence of mupirocin resistance in *S. aureus* strains has been increasingly documented. However, in our study, none of the clinical strains showed resistance related to the *mupA* or *mupB* genes. New genes influencing this resistance *in vivo* should be further investigated.

Additionally, the strains tested in our study differed in their genetic profiles, including the presence of the *bap* gene. According to Ma et al. (2021), staphylococcal Bap proteins sense environmental signals (such as pH and Ca^2+^) to modulate biofilm matrices via unknown mechanisms. Understanding the role of this protein in pathogenic bacteria could provide insights into strong biofilm formation, which may be useful in the design of antibiofilm drugs (Ma et al., 2021).

In the study by Bride et al. (2019), among MSSA strains isolated from hospitalized patients, key virulence genes included those encoding Cna adhesins (detected in 47% of MSSA isolates compared to only 3% of MRSA isolates) and the *lukS/F* genes encoding Panton-Valentine leukocidin (identified in six isolates). Similarly, this study detected the cna gene in 100% of MSSA isolates, while the *luk* genes were absent.

These findings support the idea that MSSA strains should be carefully monitored due to their considerable pathogenic potential. The presence of virulence genes may be more diverse and abundant in MSSA than in MRSA strains.

In the present study, duplicate isolations from the same patients (7* and 32*, 14** and 23**) showed differences in *bap* gene detection. Some mutants lacked *bap*, which is involved in biofilm formation and primary attachment. Both of double isolates from these same women and strains from separate patients. RAPD fingerprinting of all 33 *S. aureus* nasal carriage isolates from patients and the construction of the dendrogram of the isolates demonstrated genus similarity by applying a 4.5–5.5% polymorphic index. Genotypic evaluation requires further analyses with an increased study group and sequencing methods. Our study has certain limitations. One of the key constraints is the small sample size and the lack of direct comparison between *S. aureus* genetic diversity and clinical infection rates. It is essential to confirm the genetic similarity of isolates and assess their clonal relationships through additional molecular typing methods. Future studies should involve whole genome sequencing of representative isolates from the same patients, those associated with colonization, as well as pre- and postoperative infections to enhance understanding of transmission patterns and pathogenic potential. Nonetheless, this study has notable strengths, including comprehensive phenotypic characterization of MSSA isolates. This contributes to a better understanding of the potential etiological agents involved in pre- and postoperative breast infections within the Polish breast reconstruction patient cohort.

Given this context, continuous monitoring of the virulence of *S. aureus* from nasal carriages in preoperative procedures may be crucial to maintaining up-to-date data on these phenotypically and genotypically variable pathogenic microorganisms.

## 5 Conclusion

To our knowledge, this is the first screening study conducted in Poland that examined the virulence characteristics of MSSA strains isolated from nasal carriage in oncological patients before breast implant reconstruction following mastectomy. MSSA strains typically encode adhesion (*cna, icaADBC*), biofilm (*bap*), and regulatory (*sarA*) genes, enhancing their potential to colonize and infect surgical sites. Importantly, the pathogenic potential of MSSA is not solely dependent on antibiotic resistance but is also driven by virulence factors. Systematic preoperative nasal screening may help prevent transmission and reduce the risk of postoperative infections of *S. aureus*. Further studies should focus on assessing the relationship between nasal carriage of *S. aureus* and the development of subsequent staphylococcal infections.

## Data Availability

The datasets presented in this study can be found in online repositories. The names of the repository/repositories and accession number(s) can be found at: DOI 10.6084/m9.figshare.27910680.
