# Supplementary material for: Staphylococcus aureus nasal carriage before breast reconstruction: antibiotic resistance, biofilm formation, and virulence genes—a single center in vitro observation
Source: Front Microbiol. 2025 Jun 19;16:1610739. doi: 10.3389/fmicb.2025.1610739 (PMC12222067; doi:10.3389/fmicb.2025.1610739)
Supplement: Supplementary file 1 [file Data_Sheet_1.pdf]

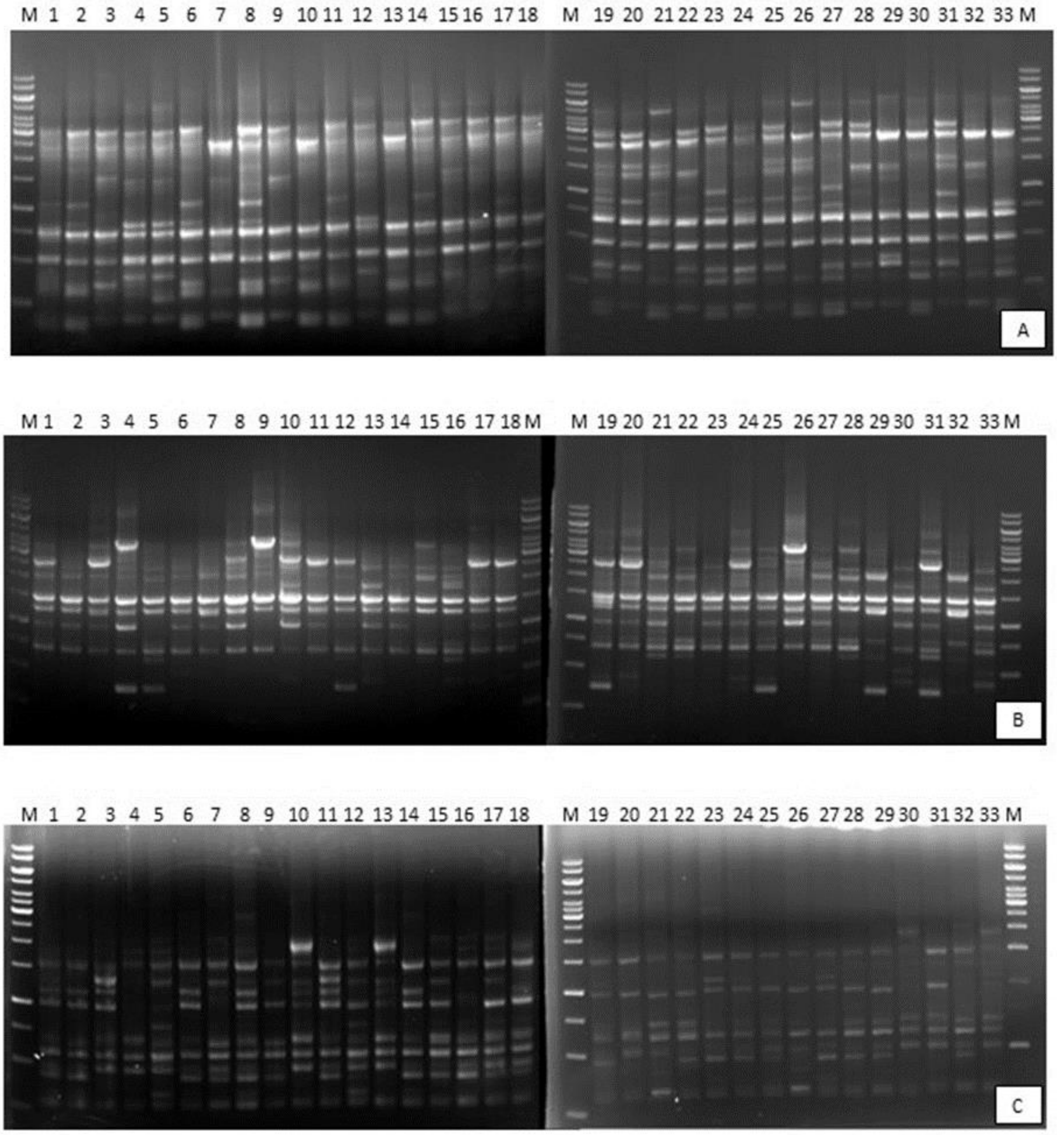

Supplementary Figure 1. RAPD-PCR fingerprinting for *S. aureus* strains, according to A) PCR-ERIC2, B) AP-PCR7, and C) AP-PCR1 primers

Abbreviation: RAPD - Random Amplification of Polymorphic DNA PCR; M – 1 kb DNA ladder; 1-33 *S. aureus* clinical strains. For the DNA fragment length of all PCR products obtained an electrophoresis on a 1.5 % (w/v) agarose gel stained with 0.5 µg/mL ethidium bromide (Merck, Germany) with using a molecular weight markers 1 kb (Thermo Fisher Scientific, UK) or 100-3000 bp (Blirt S.A., Poland), or 600-1000 bp (Blirt S.A., Poland). The DNA patterns were visualized and analyzed using the ImageLab software (Bio-Rad, US)
